# Gender Differences in Avoiding Later-Life Disability: A Life Course Perspective

**DOI:** 10.1093/geroni/igab046.776

**Published:** 2021-12-17

**Authors:** Patricia Morton

**Affiliations:** Wayne State University, Detroit, Michigan, United States

## Abstract

Identifying the early origins of adult health has underscored how experiences in the earliest stages of life can have lasting consequences. Whereas most research on the early origins of adult health has linked childhood conditions to worse health in adulthood, this study considered whether childhood conditions are associated with healthy aging. Guided by the World Health Organization’s emphasis on functional ability as a core component of healthy aging, the present study investigated the association between childhood social conditions and avoiding later-life limitations in basic and instrumental activities of daily living, referred to as disability-free status. This study also tested potential health-related and socioeconomic mediators and examined whether these life course antecedents of healthy aging vary by gender. Analyzing a sample of 9,376 adults over age 50 from the Health and Retirement Study over 10 years (2006-2016) revealed that childhood socioeconomic disadvantage reduced the odds of avoiding disability over time. For women, adult health lifestyles mediated this relationship whereas adult socioeconomic status (SES) mediated this relationship for men. Conditional indirect effects indicated that the mediational effects of body mass and education differed between men and women (i.e., moderated mediation). The direct effects of childhood and adult SES also varied by gender. These results demonstrate that the life course antecedents, especially SES, of healthy aging are distinct for men and women. Interventions should prioritize reducing early-life exposure to socioeconomic disadvantage, especially for women. Given the gendered differences in the mediating effects, midlife interventions can be tailored for men and women.

